# An extremely rare case of Rosai-Dorfman disease in the spleen

**DOI:** 10.1186/s12893-020-01014-0

**Published:** 2021-01-06

**Authors:** Xuewu Yang, Chuang Fang, Yuanpu Sha, Qi Li, Xing Zhang, Furong Du, Weijun Tian

**Affiliations:** 1 Department of General Surgery, Tianjin Medical University General Hospital, Tianjin, China; 2 Binhai Hospital of Tianjin Medical University General Hospital, Tianjin, China; 3 State Key Laboratory of Translational Medicine and Innovative Drug Development, Jiangsu Simcere Diagnostics Co., Ltd, Nanjing, China

**Keywords:** Rosai-Dorfman disease, Spleen involvement, Laparoscopic splenectomy, Histopathology, Immunohistochemistry

## Abstract

**Background:**

Rosai-Dorfman disease (RDD) is a rare, multisystemic histiocytic disorder, and commonly manifesting as lymphadenopathy in the young male. Abdominal manifestations of RDD are extremely rare.

**Case presentation:**

In August 2018, a 42-year-old man underwent an abdominal ultrasonography examination due to his weight loss of 10 kg in only three months and found a giant solid tumor was found in his spleen. Then, he was admitted to our hospital and diagnosed as a splenic mass via abdominal enhanced CT and MRI. Laparoscopic splenectomy was administrated within six days of admission due to the clear surgical indications. The pathogenesis of RDD remained poorly understood and the disease should be diagnosed based on histopathology and immunohistochemistry (IHC). The mutations in *ATM* and *NFKBIA* were observed using next generation sequencing (NGS).

**Conclusion:**

We reported a case of splenic involvement of RDD with NGS genetic testing, indicating the difficulty of making a diagnosis before surgery. This extremely rare case offers new references for the understanding of abdominal viscera RDD.

## Background

Rosai-Dorfman disease (RDD) first described in details by Rosai and Dorfman in 1969, also known as sinus histiocytosis with massive lymphadenopathy (SHML), is generally a rare benign disorder consisting of a proliferation of histiocytes with polytropic clinical manifestations, such as painless lymphadenopathy or extranodal soft tissue massed, fever, weight loss, etc. [1, 2]. RDD can occur at any age and is most commonly seen in young adults and children [3]. It is reported that up to 50% of RDD patients have extranodal manifestations, and almost every organ system can be involved, in which the involvement of gastrointestinal tract is less common [4]. Histologically, lymph nodes appear dilated sinuses and pericapsular fibrosis, infiltrate extensively into histiocytes, lymphocytes, and plasma cells [5]. Immunohistochemically, the proteins like S100 and CD68 are expressed in histiocytes [6]. In detection of RDD-related mutations, the presence of mutually exclusive *KRAS* and *MAP2K1* mutations, which regulated MAPK/ERK signaling pathway, was found in 7 of 21 RDD cases [7]. Herein, we reported a rare case of RDD in the spleen.

## Case presentation

A 42-year-old male visited to Tianjin Medical University General Hospital with a chief complaint of weight loss of 10 kg within three months. Physical examination revealed no stained-yellow sclera and no enlarged lymph nodes in the neck, underjaw, armpits, and groin. An enlarged spleen could be palpated in the left upper abdomen, but no tenderness. Abdomen ultrasound suggested that there was a huge mass in the spleen.

**Fig. 1:**
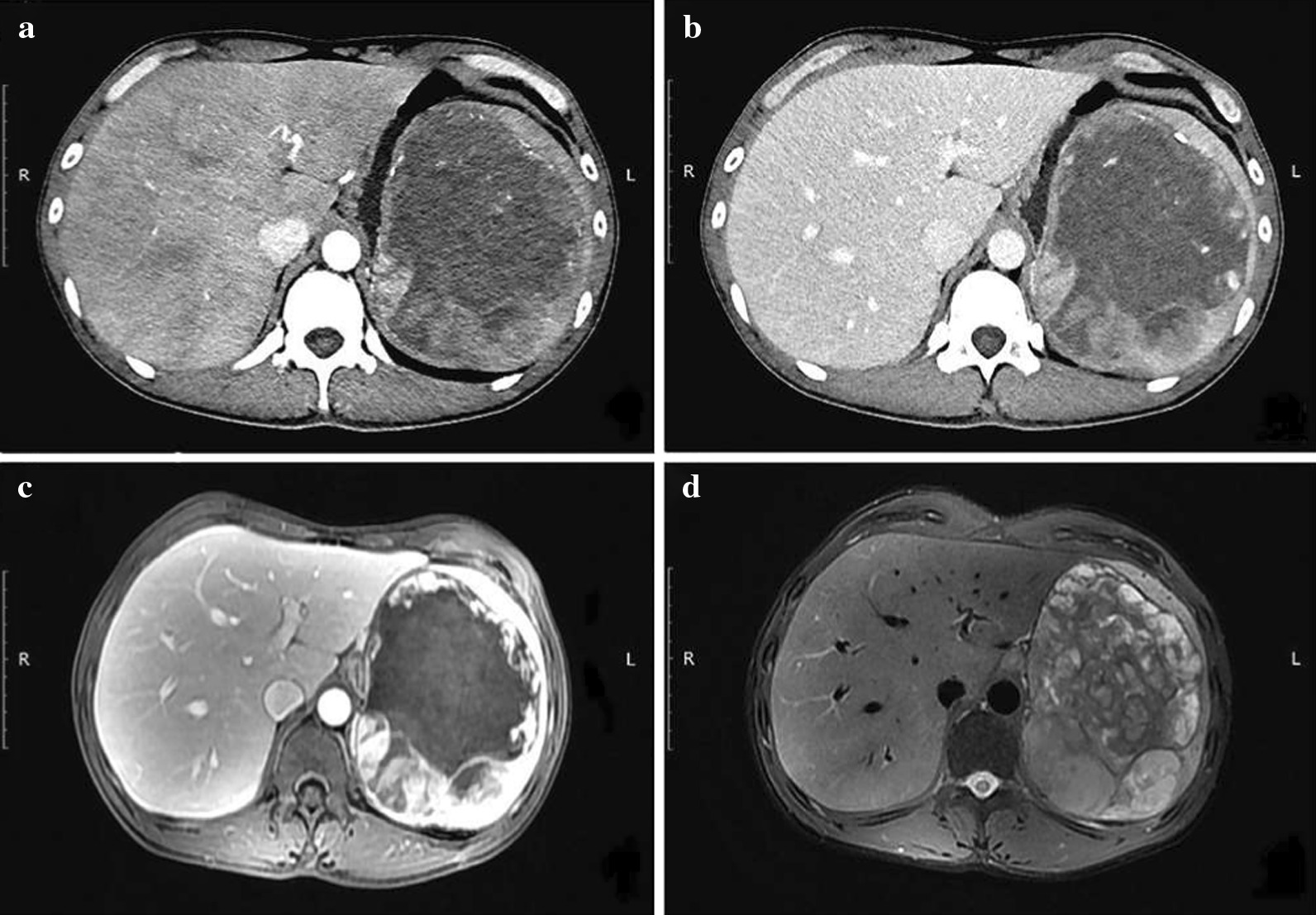
CT/ MRI-scan examinations. Enhanced CT in the upper abdomen: **a**, **b** were arterial and portal phases, respectively. Enhanced MRI in the upper abdomen: arterial phase of T2 was revealed in **c**. T1 fat-suppression images was shown in **d**

The enhanced computerized tomography (CT) and magnetic resonance imaging (MRI) were performed when the patient was admitted to our hospital for the first time. A giant solid tumor of the spleen was considered. The images of the arterial phase (Fig. 1a) and portal phase (Fig. 1b) revealed a huge low-density mass in the spleen with nodular enhancement on the margin. The separations and scattered calcifications could be seen in the spleen, with the maximum cross-section area of 12.1 × 15.1 (cm^2^). The nearby compressed, displaced tissues and organs had unclear boundaries with the greater curvature of the gastric wall. There were no enlarged lymph nodes in the abdomen and retroperitoneum, and no sign of ascites. The arterial phase of T2 (Fig. 1c) and T1 fat-suppression image (Fig. 1d) showed a huge mass with mixed signals in the spleen, including high signals in the peripheral area of T2 and diffusion-weighted imaging (DWI), and low signals in the central zone of T2 and DWI. Nodular enhancement could be found in the margin of the spleen and enlarged lymph nodes could be seen around. A blood test was used to detect the tumor markers, and the results showed that carbohydrate antigen 199 (CA199), alpha-fetal protein (AFP) and carcinoma embryonic antigen (CEA) were at a normal level but ferritin increased to 596.7 ng/ml.

**Fig. 2:**
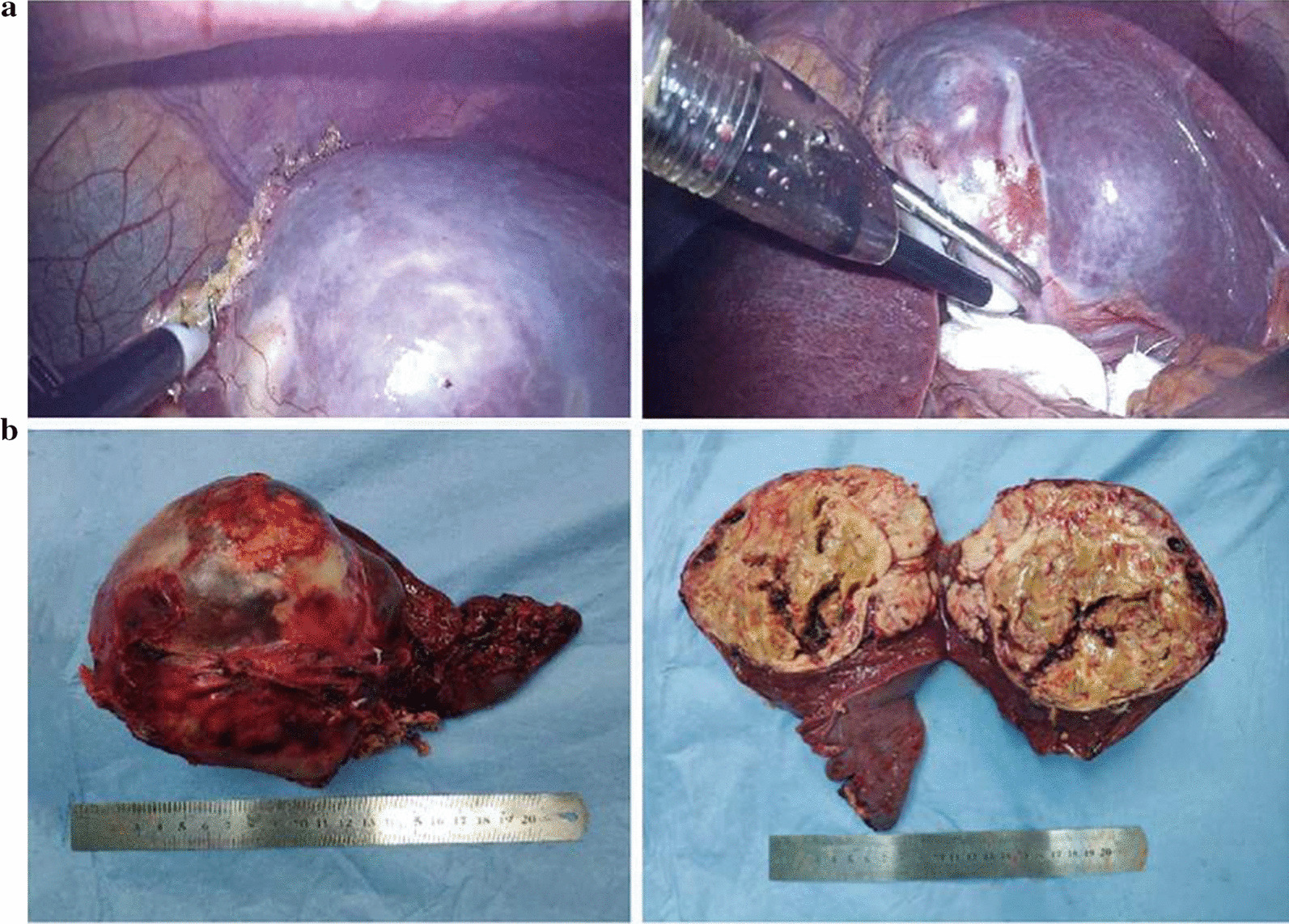
Specimen of intra-operation (**a**) and post-operation (**b**)

Laparoscopic splenectomy was performed, and the intraoperative specimen was seen in Fig. 2a. The tumor of the spleen was grayish-white and hard, with intact envelope, and the surrounding tissues were not invaded. The postoperative specimen was shown in Fig. 2b. The tumor with a diameter of about 12 cm located in the upper spleen and seemed like spherical. The section was grayish-yellow and the boundary was clear.

**Fig. 3:**
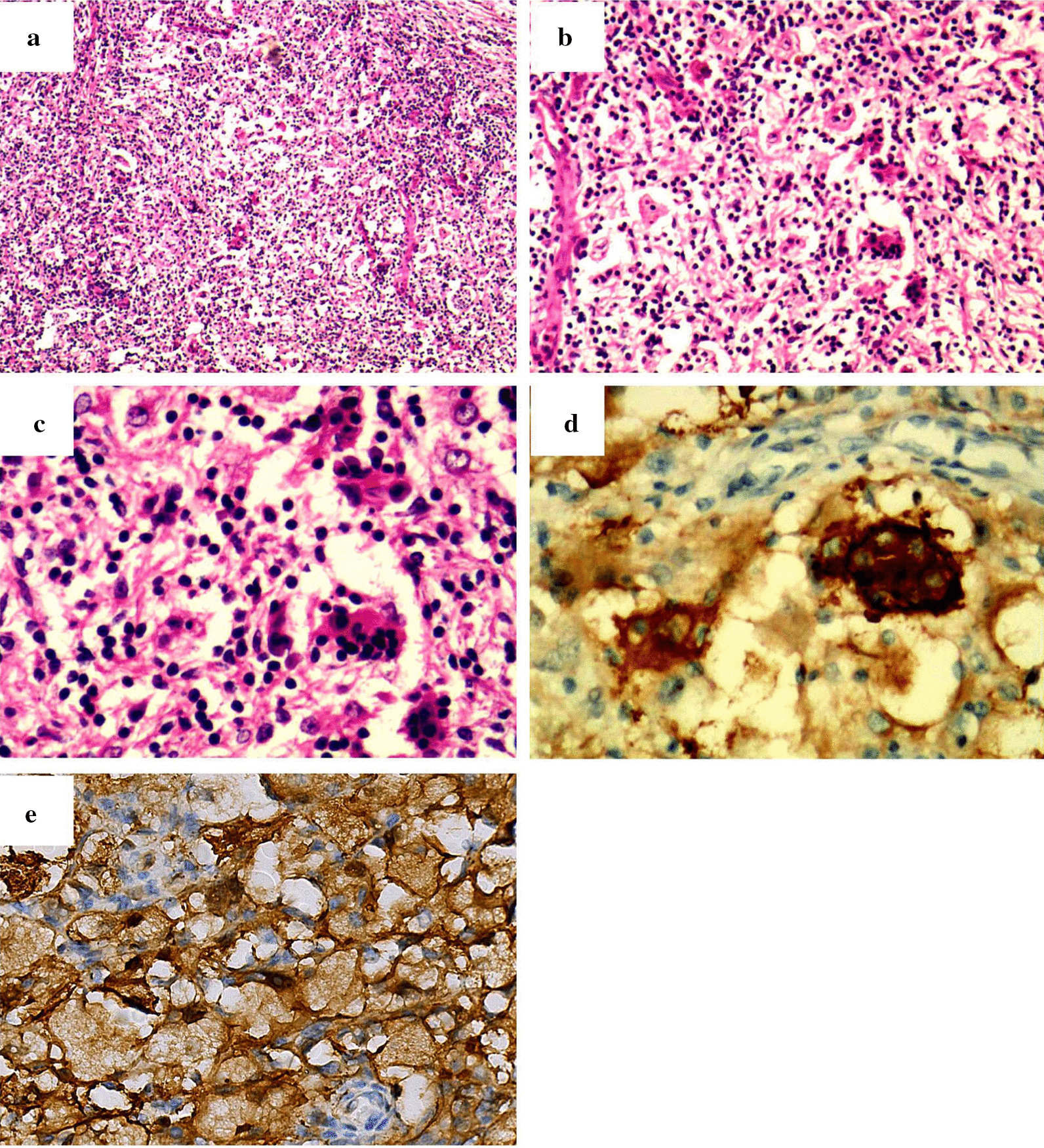
Pathologic diagnosis of the patient. Observations of cytology: **a–c** were sections with H&E staining under × 40, × 200, and × 400, respectively. Results of IHC: **d** and **e** were S100 and CD68 respectively, and both of them were positive

Postoperative pathological results via hematoxylin and eosin (H&E) indicated a cross distribution of deeply and lightly stained lesions (Fig. 3a). The deeply stained zone was mainly composed of a large number of plasma cells and lymphocytes, and interspersed in a flake-like, lightly stained area like stripes, which was known as emperipolesis. The giant pleomorphic tissue cells characterized by abundant cytoplasm, vacuoles, large nuclei, and irregular nucleus were distributed in the lightly stained area (Fig. 3b). In the cytoplasm of histiocytes, it could be observed that some lymphocytes and a small number of plasma cells were phagocytized, which was the typical phenomenon known of emperipolesis (Fig. 3c).

Immunohistochemically, S‑100 protein staining was strong positive, diffusely distributed within histiocytes in the lesion (Fig. 3d). The labeling for CD68 protein was also positive within the lesioned histiocytes (Fig. 3e). CD1a, CD21, CD23, CK, HMB45, Melanie, SMA, Desmin, EMA, CD31, CD34, Fli-1, FVIII, CD20, and CD3 were negative, and Ki67 index was about 15% in lymphocytes and plasma cells. Therefore, the patient was diagnosed as RDD.

**Fig. 4:**
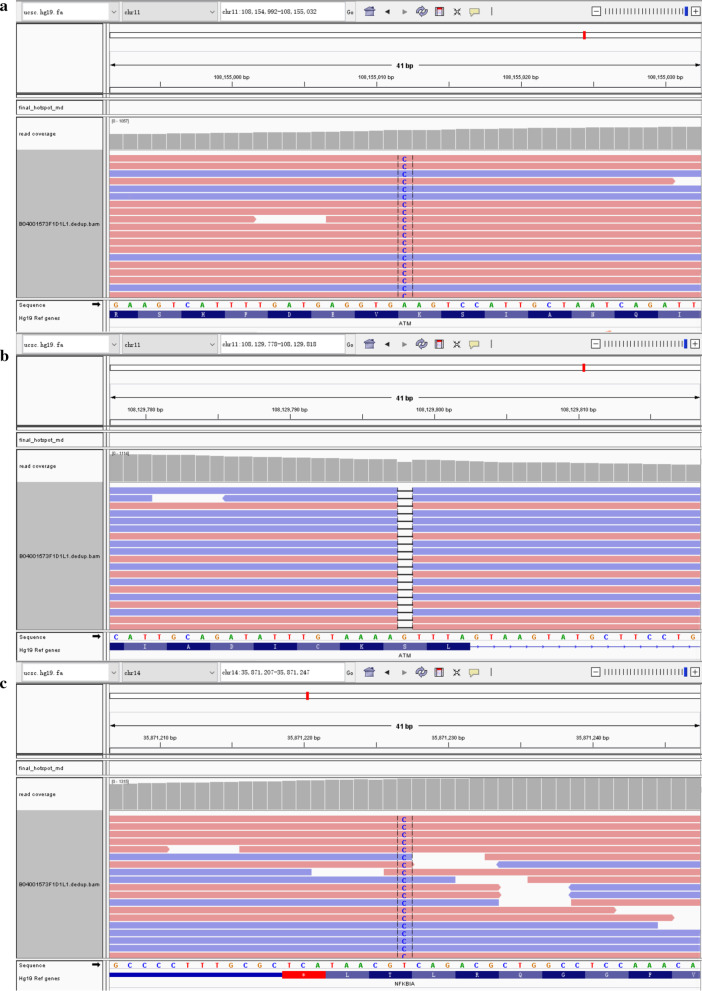
The mutations of *ATM* and *NFKBIA* in the patient with RDD. **a** Deletion mutation of *ATM*, c.2462delG, p.S821fs. **b** Missense mutation of *ATM*, c.3805A>C, p.K1269Q. **c** Missense mutation of *NFKBIA*, c.946A>G, p.T316A, respectively

To analyze the molecular character of this case and seek available options of precise treatment, a comprehensive genomic profiling with a 539-genes panel (Simceredx, Nanjing, China) was administered in biopsy specimens via NGS by DNA-based hybrid capture. NGS could detect the potential deleterious and damaging mutations (Table 1). The mutations of *ATM* and *NFKBIA*, which were genes associated with apoptosis, were identified (Fig. 4).

**Table 1:** The gene mutations detected via NGS in the patient with RDD

Gene	Mutation	Mutation abundance (%)
*ATM*	Exon16, c.2462delG, p.S821fs	13.95
	Exon26, c.3805A>C, p.K1269Q	14.80
*NFKBIA*	Exon6, c.946A>G, p.T316A	11.85
*FAT1*	Exon8, c.4516C>T, p.R1506C	14.81
*MLH1*	Exon13, c.1467A>C, p.E489D	13.87
*CUL3*	Exon4, c.382C>G, p.R128G	11.71
*RPTOR*	Intron19, c.2252+5G>C	12.42

## Discussion and conclusions

RDD is a rare histiocytic disorder with unclear mechanisms, which is easy to be misdiagnosed. The typical features of RDD are painless lymphadenectasis in the bilateral neck, usually accompanied by the symptoms such as fever, weight loss, and anemia. According to the location of lesions, RDD could be divided into three types: most commonly lymph node type, extranodal type, and mixed type [8]. As an extranodal subtype, abdominal viscera manifestations are extremely rare in RDD [4].

RDD is still a disorder with unclear etiology. Some researchers thought that RDD was related to virus infections such as human herpesvirus (HHV), human parvovirus B19, and Epstein-Barr virus [9, 10], and human immunodeficiency virus (HIV) and human herpes virus (HHV) had been detected in RDD diseased tissue [11, 12]. Previous studies suggested the pathogenesis of RDD might be associated with monocyte/macrophage colony-stimulating factor (M-CSF) which led to the production of immunosuppressive macrophages and signal transduction by the genome alterations and homeostasis interference [13]. However, it remained controversial about the association of IgG4 with RDD [9]. RDD is considered being an autoimmune disorder due to its frequent coexistence with immune-mediated diseases including asthma, systemic lupus erythematosus, rheumatoid arthritis, and hemolytic anemia [14–16]. In our case, the absolute value of monocytes increased, which appeared to support this opinion partially.

Emperipolesis and storiform structure are the hallmark characters of RDD, however, emperipolesis is not pathognomonic [17–19]. Therefore, more examinations including IHC and special staining need to be performed to exclude other diseases. The RDD cells are positive for phagocytic markers including S100 and CD68, but not for markers such as CD1a and CD23 [3]. The patient was confirmed as RDD via both histology and IHC techniques. There were two splenic RDD reported before, one was confirmed by imaging [4], the other was diagnosed via pathology [20]. H&E staining of the latter patient showed that trilineage extramedullary hematopoiesis was found to be extensive, which also existed in peripheral lymph nodes. Meanwhile, multifocal S-100 positive histiocytic aggregates were also presented.

The literature review indicates 20% of RDD patients can be self-healing, 70% are in stable condition, only 10% arise have aggressive local lesions or systemic disease [21]. RDD could be fatal for a very small percentage of patients if the giant enlarged lymph nodes compress vital organs, with the combination of mass formation and cellular infiltration [22, 23]. For symptomatic RDD patients or those with involvement of critical organs, the treatments including surgery should be given [9].

The MAPK/ERK is a critical regulatory pathway in various cellular processes, such as cell proliferation, differentiation, survival, and apoptosis [24]. The mutations in *KRAS* and *MAP2K1* genes can activate MAPK/ERK, which are considered to be associated with RDD [7]. In 9 RDD cases undergoing whole exome sequencing, 3 harbor *NOTCH1* gene mutations involving in cell apoptosis [25]. Children with RDD have a similar phenotype to autoimmune lymphoproliferative syndrome which is associated with Fas-mediated apoptosis [26]. *ATM* and *NFKBIA* genes also play important roles in regulating apoptosis [27, 28]. We speculate that mutations in apoptosis-related genes which could lead to abnormal apoptosis of histiocytes and lymphocytes may be associated with RDD. However, it needs further confirmation in a larger cohort.

To the best of our knowledge, this is the second spleen occurring RDD case with specific proof via histology and IHC, but the first splenic RDD case with analysis of gene mutations. Our findings suggest the difficulty of making a diagnosis before surgery and provide new references for the understanding of abdominal viscera RDD.

## Abbreviations

AFP: Alpha-fetal protein
CA199: Carbohydrate antigen 199
CEA: Carcinoma embryonic antigen
CT: Computerized tomography
DWI: Diffusion-weighted imaging
H&E: Hematoxylin and eosin
HHV: Human herpes virus
HIV: Human immunodeficiency virus
IHC: Immunohistochemistry
M-CSF: Monocyte/macrophage colony-stimulating factor
MRI: Magnetic resonance imaging
NGS: Next generation sequencing
RDD: Rosai-Dorfman disease
SHML: Sinus histiocytosis with massive lymphadenopathy

## Availability of data and material

Data supporting the present findings can be obtained, after obtaining permission from the Tianjin Medical University General Hospital. The data can be obtained by corresponding author.
